# Small-scale alpine topography at low latitudes and high altitudes: refuge areas of the genus *Chrysanthemum* and its allies

**DOI:** 10.1038/s41438-020-00407-9

**Published:** 2020-11-01

**Authors:** Xi Chen, Haibin Wang, Xiaodong Yang, Jiafu Jiang, Guopeng Ren, Zijuan Wang, Xiaodong Dong, Fadi Chen

**Affiliations:** 1 State Key Laboratory of Crop Genetics and Germplasm Enhancement, Key Laboratory of Landscaping, Ministry of Agriculture and Rural Affairs, College of Horticulture, Nanjing Agricultural University, 210095 Nanjing, China; 2 College of Agriculture and Biological Sciences, Dali University, 671003 Dali, China; 3 Institute of Eastern-Himalaya Biodiversity Research, Dali University, 671003 Dali, China

**Keywords:** Plant ecology, Plant evolution, Biodiversity, Genetic markers

## Abstract

Cultivated chrysanthemum (*Chrysanthemum morifolium* Ramat.) is an economically important ornamental plant species grown worldwide. However, the origin of the genus *Chrysanthemum* remains unclear. This study was conducted in the Hengduan Mountains, Yunnan Province. We took advantage of a special geographic region where the southernmost species of *Ajania* and the highest altitude population of *Chrysanthemum indicum* coexist to investigate their evolutionary origins. Diversity analysis of 9 populations of 5 species that came from 3 genera was carried out based on morphological traits and SRAP markers. Furthermore, topographical and ecological analyses and surveys of the vegetation communities in the plots were carried out for correlation analysis, and past data were used to reconstruct the ancient topography and vegetation to estimate the migration path and divergence time. We found that *Chrysanthemum* and *Ajania* were closely related based on the smooth transition states among marginal female florets and their common pollination system. The genetic relationship between *Phaeostigma* and *Chrysanthemum* was relatively distant, and *Ajania* was between them. Low light intensity and relatively humid habitats may be driving the elongation and evolution of marginal female florets. We found that *Chrysanthemum* and related genera were largely restricted to stony topographies at an altitude of ~3000 m.a.s.l. and in specialized alpine coniferous (*Pinus*) and broad-leaved (*Quercus*) mixed forest marginal communities. These stony topographies have become ecological islands of refuge for these species in the current interglacial period. The Hengduan Mountains play a key role in the evolution, divergence, and survival of *Chrysanthemum* and its allies.

## Introduction

Cultivated chrysanthemum (*Chrysanthemum morifolium* Ramat.), as one of the most economically important ornamental plants grown worldwide^1^, has been popular among horticulturists and botanists for a long time^2^. Most research on cultivated chrysanthemum has focused on breeding, disease control, regulation of horticultural characteristics, and their molecular mechanisms^3–9^. There are relatively few studies on the evolutionary relationships between the genus *Chrysanthemum* and its wild relatives, especially with regard to biogeography and ecology, for which the origin of chrysanthemums would be useful information.

*Chrysanthemum* has 41 species that are widely distributed in Asia (Mongolia, Russia, China, Japan, and Korea) and eastern Europe^10^. There are approximately 21 species distributed in most parts of China, except Tibet and Northwest China, and most of these species grow in humid areas at low and medium altitudes.

*Ajania* Poljakov (Anthemideae, Asteraceae) is a sister group closely related to *Chrysanthemum* that can be distinguished by its disciform capitula; *Chrysanthemum*, on the other hand, has radiate capitula^11^. *Phaeostigma* established by Muldashev^12,13^ was a genus circumscribing the Chinese endemic species formerly described under *Ajania* based on palynology and morphology^12,14^, and this view was supported by molecular phylogeny^15^. *Ajania* and *Chrysanthemum* are not easy to distinguish clearly based on the nuclear ribosomal ITS, chloroplast DNA sequences or single-copy nuclear genes^16,17^. Hybrids between these taxa are easy to obtain, and their chromosomes are similar to each other. Ohashi and Yonekura^18^ included *Ajania* in *Chrysanthemum*. However, the results of phylogenetic comparisons among different samples and gene sequences are divergent^16,17,19^. The characteristics of marginal female tubular florets in *Ajania* can be significantly different from those of ray florets in *Chrysanthemum*, and more importantly, the geographical distributions of the genera *Chrysanthemum* and *Ajania* in China have obvious differences^20^. According to the records of the Flora of China and the Chinese Virtual Herbarium, *Chrysanthemum* is usually distributed to the south of China’s 500-mm isoline, whereas *Ajania* is mainly distributed to the north of China’s 800-mm isoline, and both are distributed between the two isolines. In addition, *Chrysanthemum* is usually distributed below 3000 m.a.s.l. Hence, the evolutionary relationship and origin of the two genera are still unclear. In particular, the ancestral floret characteristics being the same as those of marginal female florets, either tubular florets or ray florets, is a critical issue.

The evolution of species is driven by the environment. To explore the key issues of evolutionary history, such as the region of origin, divergence time, radiation path, and distribution range, it is necessary to combine fossil^21^, phytogeographical^22^, ecological^23,24^, and other evidence to obtain reasonable and accurate results. In these aspects, there is a paucity of research on *Chrysanthemum*, *Ajania*, and their allies (but see Li et al.^25^), especially on the phytogeography and ecology within their areas.

Mountains contribute disproportionately to the terrestrial biodiversity of Earth^26^. Mountain regions are unusually biodiverse, with rich aggregations of small-ranged species that form centers of endemism^27^. Owing to the diversification of habitats and climates, mountains have become refuges and centers of diversity for many species^26,28–31^. The Hengduan Mountains, located in southwest China, are a hotspot of biodiversity^32^. The Hengduan Mountains are home to many temperate plant species and are a center of distribution and speciation for many alpine taxa^33–35^.

The research site of this study was in the Erhai Lake Range, Dali, Yunnan Province, which is located in the southern margin of the Hengduan Mountains. The latitude here is between N25.482° and N26.543°, and the lake-level elevation is 1972 m.a.s.l., whereas the surrounding mountains are almost more than 3000 m.a.s.l. (the highest elevation is more than 4000 m.a.s.l.), which is a typical low-latitude and high-altitude area. As an endemic species of this area, *Ajania sericea* (*A. sericea*) is the southernmost recorded species of the genus *Ajania* in China at present, residing to the south of China’s 800 mm isoline. This special geographical distribution has important significance for the study of biogeography and evolution. However, only one specimen has been collected worldwide, in the collections of Harvard University Herbaria and Libraries, since it was discovered by J. M. Delavay in Yan-in-chan in 1887, and there is a paucity of research on this species. In addition, we found several populations of *Chrysanthemum* distributed at altitudes over 3000 m.a.s.l. here.

In this study, the main ranges around the Erhai Lake Basin were surveyed comprehensively in different periods for 3 years (2016–2018), and samples of *Chrysanthemum* and its allies (including *A. sericea*) were collected along with geomorphologic and geographic information on their habitats. In addition, we surveyed the plant community of the habitats for ecological analysis. Morphological diversity and genetic diversity were analyzed based on traits and sequence-related amplified polymorphism (SRAP) markers, respectively, and the special geographical distribution was documented using geographic information systems (GIS); correlation analysis was performed by combining topography and ecology. Last, the paleogeomorphology, paleovegetation and paleoclimate^36^ during the upper Eocene and the last glacial of the Quaternary in this study area were reconstructed based on previous studies of paleopalynology and geology to deduce the potential evolutionary relationship and history of the two genera. Our results will provide a new research methodology and perspective for the study of the evolutionary history and origin of *Chrysanthemum* and its allies through the in-depth study of biogeography and ecology.

## Materials and methods

### Study site

The research area was the mountains, river valleys, and basins around Erhai Lake, from N25.482° to N26.543° and E99.895° to E100.524°, within a rectangular range of approximately 120 × 65 km in Dali, Yunnan Province, China. Fieldwork analysis was conducted during May–December of 2016–2018; we searched for the species of the genus *Chrysanthemum* and its allies throughout the study area to obtain comprehensive distribution data. Moreover, the main vegetation types in this study area were recorded to understand vegetation characteristics at different altitudes. Furthermore, for each plot in which target species were found, plant community and topography data were collected in plots for correlation analysis.

### Sampling and data collection

Three replicate plots (10 × 10 m) were randomly established within each plot^37^, and each replicate plot was spaced 10–50 m apart^38^. The number of target plants in each plot was recorded as the abundance (AB). The species richness of the tree layer (TS, height> 2 m, highly lignified), shrub layer (SS, height <2 m, lignified), and herb layer (HS, height <0.5 m) and the total richness (*R*, where *R* = TS + SS + HS) were separately recorded within each plot. TS was determined to the species level because of the significance that presenting detailed descriptions of tree species composition would have for this study, and SS and HS were identified to the genus level. Photos were taken for comparison with herbarium specimens if a species could not be identified in the field^39^ or had great significance to this study. The percentage cover^40,41^ of the tree layer (TLC), shrub layer (SLC), and herb layer (HLC) were estimated based on the surface area. The surface rock coverage (SRC) as a topographical factor in this study was estimated by the following formula:

$${\mathrm{SRC}} = \frac{{0.7\mathop {\sum }\nolimits_{i = 0}^n Li \times wi}}{P}$$

(*L*(m): maximum length of rock; *W* (m): maximum width of rock; 0.7: correction factor based on the empirical value obtained after 20 different measurements; *p*: plot area (100 m^2^)).

To assess the morphological diversity, based on morphology, 44 qualitative traits (Table S2) were recorded and coded with different levels during the flowering period. We randomly collected 10 replicate plant samples (0–5 samples from each plot) from three replicate plots to measure 12 quantitative traits for principal component analysis (PCA): plant height (PH), stem diameter (SD), maximum leaf length (MLL), maximum leaf width (MLW), petiole length (PL), compound corymb diameter (CCD), capitulum diameter (CD), ray floret length (RFL), involucre length (IL), marginal female tubular floret length (FTL), tubular floret length (TL), and achene length (AL).

To assess the genetic diversity, based on SRAP markers, genomic DNA was extracted from the leaves of each species using a slightly modified version^42^ of the Murray and Thompson^43^ hexadecyltrimethylammonium bromide (CTAB) method. The SRAP method followed the protocol described by Zhang et al.^44^, and analysis was performed as described by Li and Quiros^45^. Analysis of genetic relationships was performed with NTSYS-pc 2.1 based on UPGMA (unweighted pair-group method with arithmetic means). The sequences of the forward and reverse SRAP primers used in this study are shown in Table S1.

We determined geographical coordinates and altitude (m.a.s.l.) of each sampling area by GPS. To understand the pollination traits, we recorded the florescence and took photos of the pollinating insects of each target population as evidence. The current main vegetation types of the study area were recorded within altitude gradient intervals; according to the previous data of paleopalynological and geological research^46–52^, the ancient topography and vegetation communities were reconstructed based on time nodes. These data were used to deduce the potential evolutionary relationship and estimate the divergence time between *Chrysanthemum* and its allies via comparison with the current situation.

### Statistical analyses

The digital elevation model (DEM) was based on the Shuttle Radar Topography Mission (SRTM 3, NASA and NIMA, USA) data. We used ArcMap10.5 (Esri USA) to extract the aspect, slope^53^, and topographic position index for GIS analysis^54^. To more intuitively and accurately show topographic features, we made DEM profiles for each population. Since most of the mountains in this area are north-south oriented, we adopted east-west cross sections that were parallel to the latitude. In addition to the coefficient of variation (CV), the Shannon-Weaver diversity index (*H’*)^51,55^ was calculated based on qualitative traits using the following formula:

$$H^\prime = - \sum Pi\,{\mathrm{ln}}Pi\,\,\,Pi = \frac{{Xi}}{P}$$

(*P*: populations = 9, *Xi*: the number of code values within the *i*th level for a qualitative trait. Level: the average value (*A*) and standard deviation (S) of all codes of a certain trait were calculated and then divided into 10 levels, from the first level (*X* < A–2S) to the tenth level (*X* > *A* + 2S), where each 0.5S is a level)

The differences between two populations of the species that live in two different plots, based on 11 quantitative morphological indexes, can be assessed by *T*-tests. To clearly understand the differences among 11 quantitative morphological indexes that form different species and populations, we conducted a principal component analysis (PCA) in Origin 2019 (OriginLab, USA). To account for potential correlations between the target species abundance, plant community structure and topography that could explain differences in the distribution patterns of *Chrysanthemum* and its allies, a correlation matrix^56^ was established using R 3.6.1 (R Core Team, 2016).

## Results

### Altitude and topographic features based on field work

**Fig. 1:**
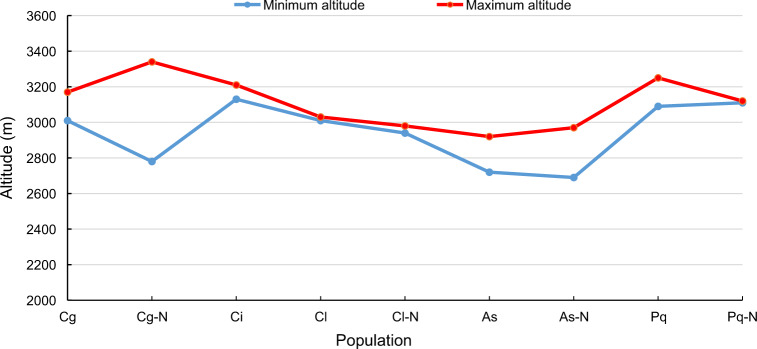
Distribution altitude range of the nine populations

**Fig. 2:**
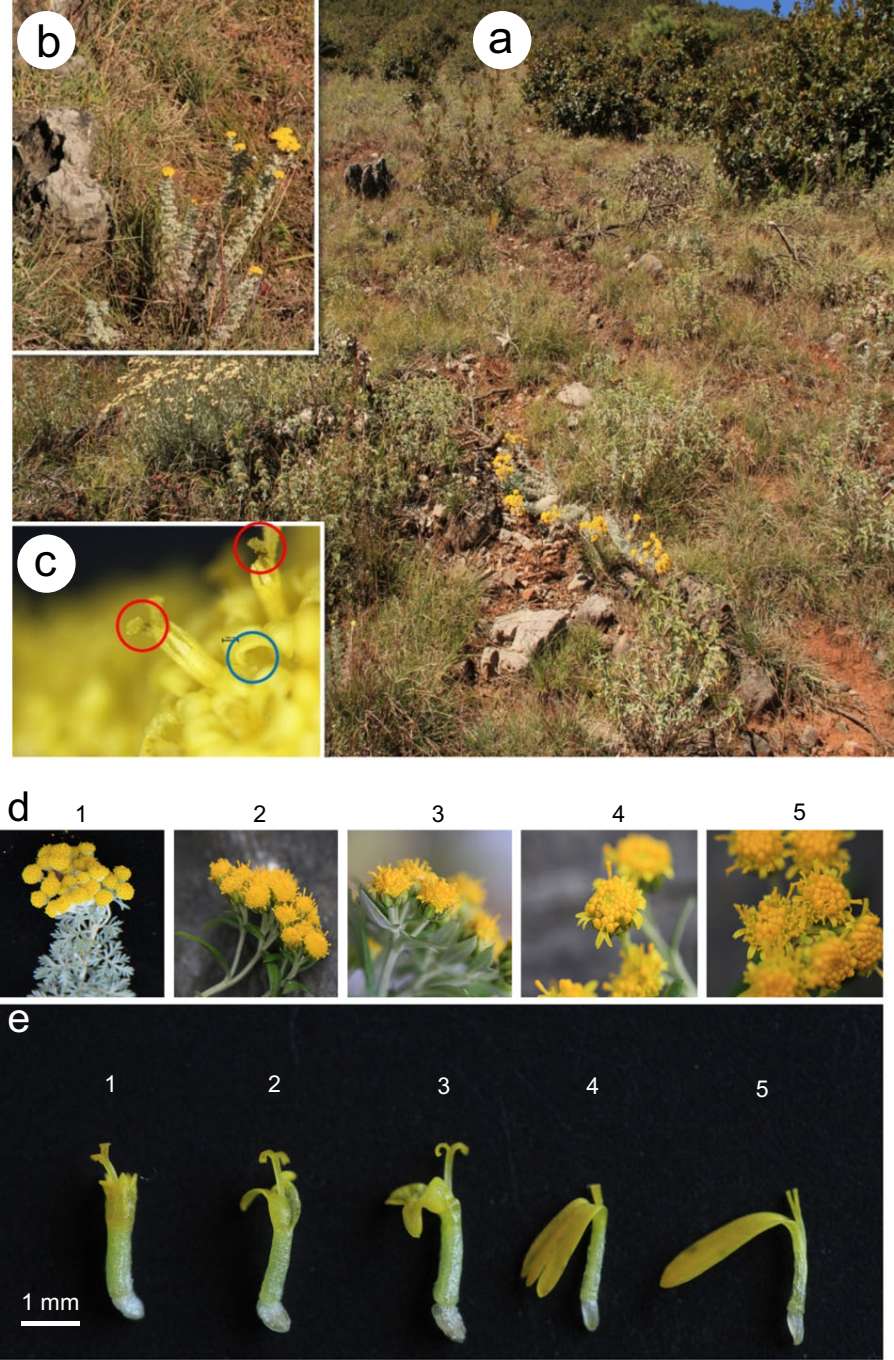
Stony habitats, microphotographs of corolla and capitula for *Chrysanthemum* and its allies. **a** The habitat of *Ajania sericea* in the As population, (**b**) the surface rocks, (**c**) microphotographs of the glandular trichomes on the corolla (blue) and brownish style branches (red). **d** The diversity of capitula and (**e**) marginal female florets (ray florets) between the As (1) and Cg-N (2, 3, 4, and 5) populations. We chose fully mature female florets with Y-type stigmas (1, 2, and 3) to exclude the possibility of incomplete development and immature female florets (4 and 5) for comparison

In the study area, three species of *Chrysanthemum*, one species of *Ajania*, and one species of *Phaeostigma* were found in nine independent small-scale alpine areas (Table 1 and Fig. 1). We found that in the study area, *Chrysanthemum* and its allies were all distributed in high-altitude mountains and displayed a special pattern, being mainly distributed around an elevational range of 3000 m.a.s.l. ±300 m.a.s.l. The altitude range of most populations was relatively narrow, except for Cg–N (Fig. 1). Moreover, the five species were separated from each other by geographical isolation, and there was no overlap in the distribution of the two species in a plot. We observed similar topographic features with large numbers of surface rocks or screes and a thin soil layer in the nine plots (Fig. 2a, b and Fig. S1) in which *Chrysanthemum* and its allies were observed in this area, which suggests that they have low waterlogging tolerance.

**Table 1:** Populations, distributions, and florescence of *Chrysanthemum* and its allies in study area

Taxon	Population	Distribution	Florescence
*Chrysanthemum glabriusculum* (W. W. Smith) Handel–Mazzetti	Cg	N 25.695° E 100.108°	Late Oct.–Late Dec.
	Cg–N	N 25.727° E 100.094°	Mid Oct.–Early Dec.
*Chrysanthemum indicum* Linnaeus	Ci	N 25.974° E 100.360°	Early Nov.–Mid Dec.
*Chrysanthemum lavandulifolium* var. *tomentellum* (Handel–Mazzetti)	Cl	N 25.556° E 100.435°	Early Nov.–Late Nov.
Ling et Shih	Cl–N	N 25.567° E 100.430°	Early Nov.–Late Nov
*Ajania sericea* Shih	As	N 26.095° E 100.118°	Early Oct.–Mid Nov.
	As–N	N 26.184° E 100.054°	Early Oct.–Mid Nov.
*Phaeostigma quercifolium* (W. W. Smith) Muldashev.	Pq	N 25.853° E 100.045°	Late Sep.–Mid Nov.
	Pq–N	N 26.503° E 100.001°	Late Sep.–Early Nov.

### Diversity and principal component analysis of morphological characteristic data

Although the altitude and topography were similar, the 9 populations showed high morphological diversity based on the 44 qualitative traits (Table S5). The qualitative traits with high diversity mainly focused on the shapes and divisions of leaves, the presence of trichomes on stems and leaves, colors of the scarious margins of phyllaries, ray florets, and marginal female tubular florets. The diversity index of upper stem leaf shapes was the highest (*H*′ = 1.58) (Fig. S4, Table S5). Subsequently, we found that there were sessile glands (Fig. 2c, Fig. S1) on the corollas^57,58^ of all five species (*H*′ = 0.00); in particular, this trait was reported for the first time for *A. sericea*, *Chrysanthemum glabriusculum*, and *Chrysanthemum lavandulifolium* var. *tomentellum*. Notably, *A. sericea* exhibited morphological characteristics that suggested that it has very high drought tolerance in comparison to other species. These characteristics included densely packed trichomes all over the plant, completely subdivided leaves, and narrowly elliptical ultimate segments.

*Phaeostigma quercifolium* was significantly different from other species, exhibiting qualitative traits that were different from those noted in previous studies, such as a subshrub posture, the presence of cicatricles, lateral expansion of the petiole base, yellow-white tubular florets, an absence of rhizomes, and annular lobules at the petiole (Table S5). However, brownish style branches were found in both *P. quercifolium* (Fig. S1a) and *A. sericea* in this study (Fig. 2c), which was a key trait that separated *Phaeostigma* from *Ajania* according to Muldashev^12^.

Ray florets of the Cg–N population showed a high diversity of lengths and a range of characteristics. For example, plants that grew on open stony slopes had shorter ray florets and an obvious tri-denticulate apex, which were similar to the Cg population that grew in dense shrubs (Fig. 2d (2–5)). We compared the morphology between the marginal female florets (*A. sericea*, As population) (Fig. 2e (1)) and the ray florets (*C. glabriusculum*, Cg–N population) (Fig. 2e (2–5)), and obvious transition states in the morphology were observed (Fig. 2e (1–5)). Notably, the half-tubular female florets (Fig. 2e (2)) indicated that the ray florets of *Chrysanthemum* might have originated from the splitting and elongation of the marginal female tubular florets that came from *Ajania*, which was an important piece of evidence for determination of the evolutionary relationship between *Ajania* and *Chrysanthemum*.

There were no significant differences in most quantitative traits between the two different populations of the same species (*P* < 0.05). The main significant differences came from the PH. However, the RFL of the Cg-N population was significantly higher than that of Cg population (*P* = 0.014, Table S6), which again supported the differences seen in the qualitative traits.

**Fig. 3:**
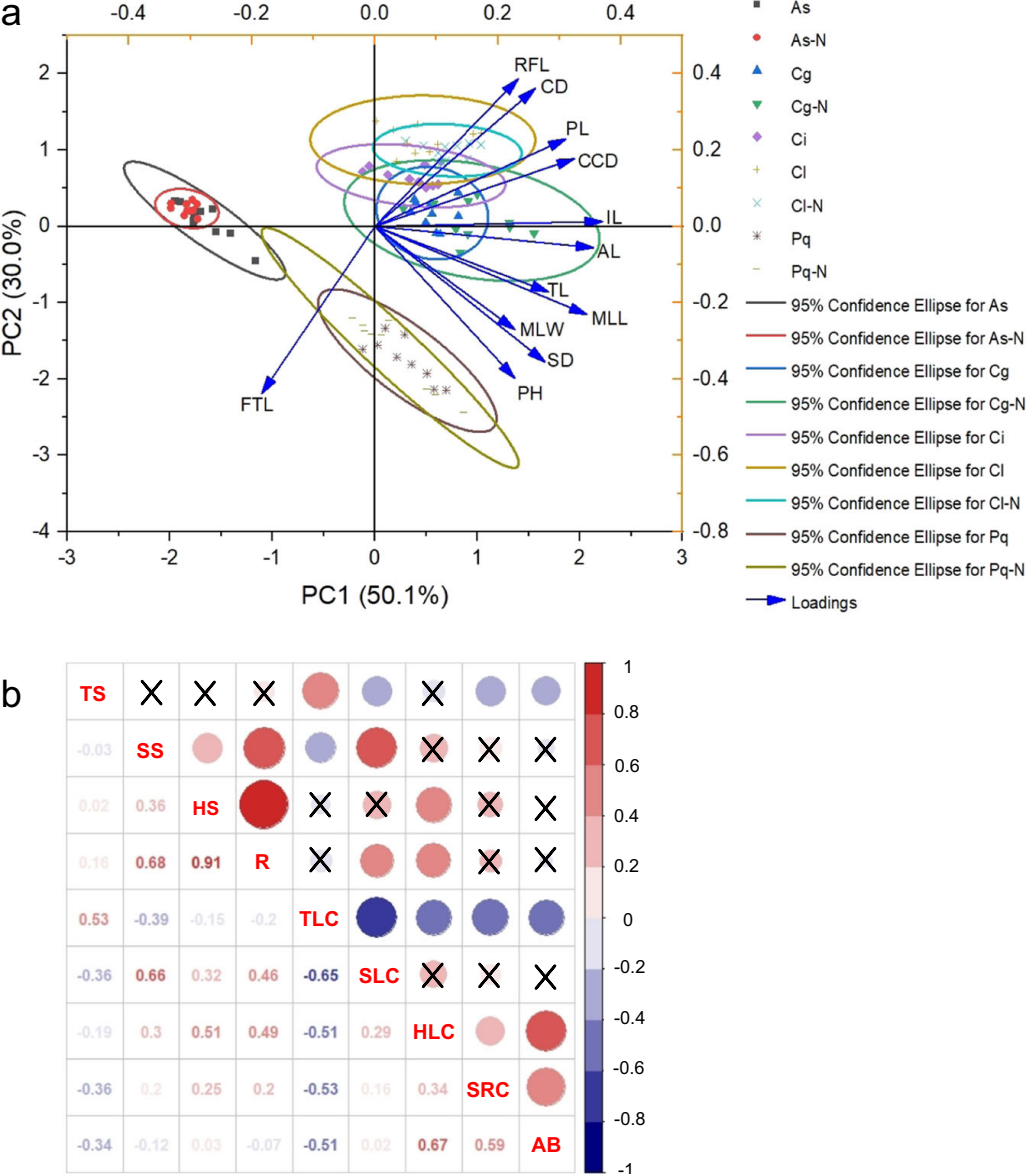
Principal component analysis (PCA) and correlation analysis. **a** PCA of 12 quantitative traits from 9 populations. **b** Correlation matrix for 27 plots; “×” marks coefficients that were not significant at the α = 0.05 level

PCA with 12 quantitative traits from 9 populations generated 3 clusters, namely, the *Chrysanthemum* cluster, the *Ajania* cluster and the *Phaeostigma* cluster (Fig. 3a). For the first principal component, almost all quantitative traits were positively correlated except FTL. We suggest that plant type and organ size constitute the factors for PC1. Since the second principal component was positively correlated with CD, CCD, and LFL, we suggest inflorescence as the significant factor for PC2. *Chrysanthemum* populations had more significant inflorescences than the other two genera according to the scatter from PC2, and *P. quercifolium* had larger plant types than PC1. *A. sericea* was significantly smaller than other species.

### Genetic diversity and cluster analysis

**Fig. 4:**
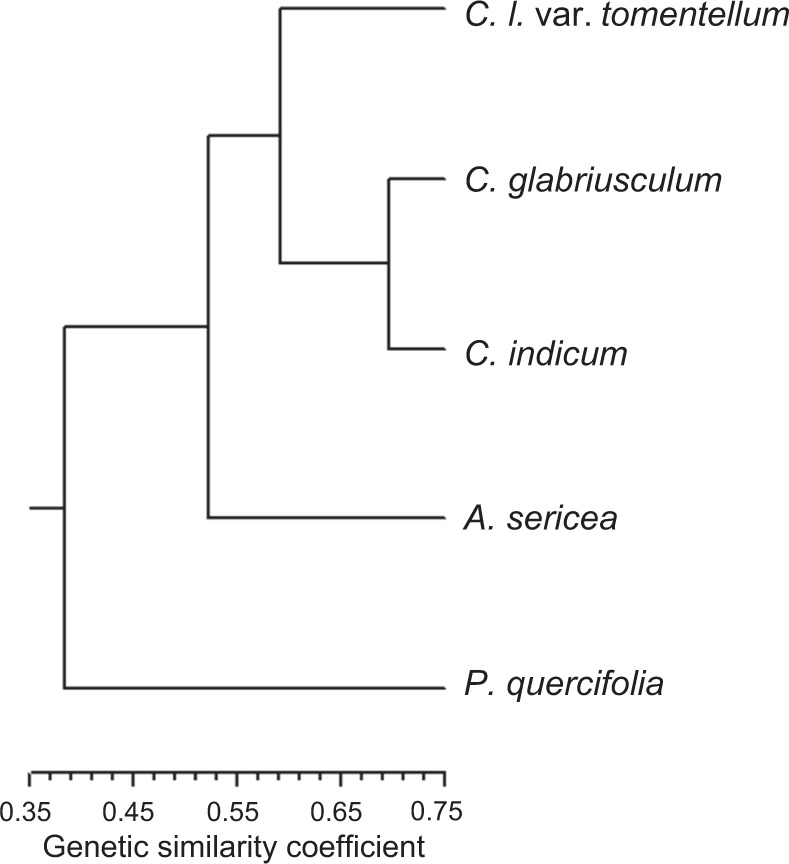
Dendrogram of 5 species genotypes from cluster analysis based on SRAP markers

From the 24 appropriate SRAP primer combinations, a total of 276 reliable bands were scored, of which 269 (97.46%) were polymorphic (Table S2, Fig. S5). The genetic similarity coefficient among the five genotypes ranged from 0.38 to 0.70, with an average of 0.55 (Fig. 4). The largest genetic distance was recorded between *P. quercifolia* and *C. l*. var. *tomentellum*; the lowest was between *C. glabriusculum* and *C. indicum*. The dendrogram of genetic relationships among genotypes revealed by the UPGMA method is presented in Fig. 4, and three main clusters were generated in this tree. *P. quercifolia* and *A. sericea* formed a separate cluster, and three species of *Chrysanthemum* clustered into another group. The genetic relationship between *Phaeostigma* and *Chrysanthemum* was relatively distant, and *Ajania* was between them. This result is similar to the PCA result (Fig. 3a) based on morphology. Therefore, there was homogeneity between morphological diversity and genetic diversity.

### Topographic analysis based on GIS

**Fig. 5:**
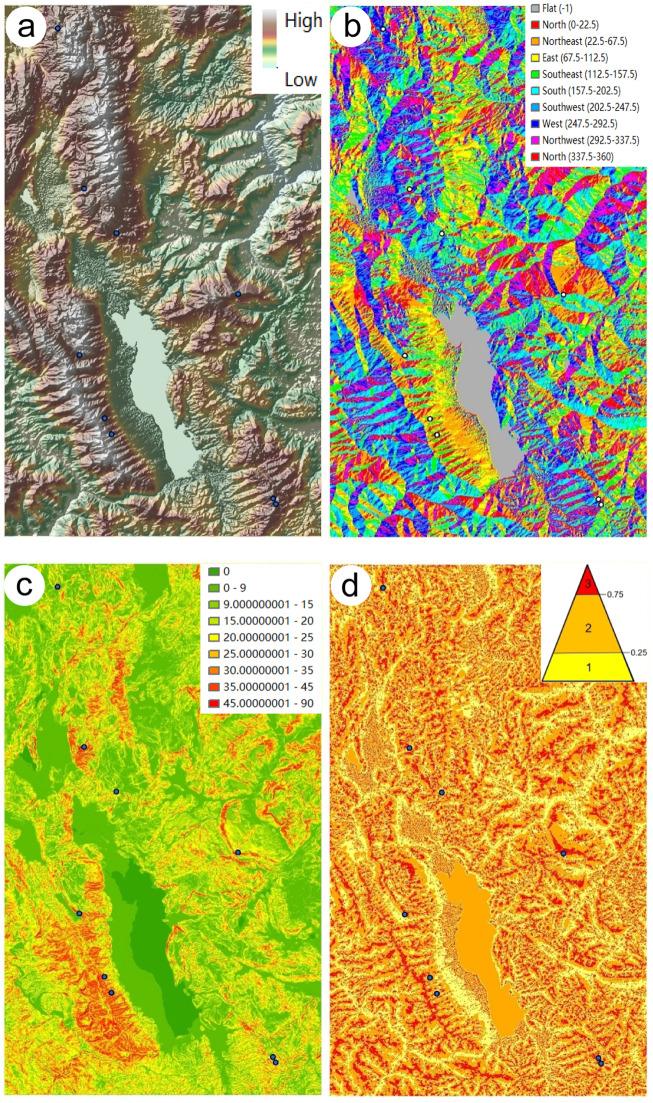
The topographic information of nine plots determined by GIS. (**a**) elevation, (**b**) aspect, (**c**) slope, (**d**) topographic index

Our results show that there were no *Chrysanthemum* species or their allies distributed in the valleys and basins that are found in low-altitude regions (Fig. 5a); the 9 populations were all in the mountains, with the highest peak at over 3000 m.a.s.l. Most of the populations were located on the southern, southeastern and southwestern aspects except for Cg-N, which was on the northern aspect, with less light (Fig. 5b). In contrast, two populations of As were on the southern aspect (aspect values: 183.52, 193.06), with the longest light time (Fig. 5b, Table S4). In addition, Cg and Cg–N were very close to each other, with similar elevations and topographies, and the only difference was that Cg was on the eastern aspect (67.75), with more direct light and relatively drier habitats. We hence argue that *Chrysanthemum* and its allies were most likely heliophilous, especially *Ajania*, and the northern aspect with lower light intensity and relatively humid habitats may be the key factor driving the elongation and evolution of marginal female florets. The slopes of habitats for all populations were more than 15°, and most of them were more than 30° (Fig. 5c, Table S4). However, in the actual survey, the microtopography of each sampling area was relatively complex, and protruding large rocks and cliffs were often present, which made the slope value larger. Through the topographic position index analysis, we found that all populations lived at high topographic positions in the mountains (index ≥ 2) (Table S4), and most were on the tops of the mountains (Fig. 5d).

The small-scale topographic features of the nine populations displayed two modes based on the cross section (Fig. S2). One mode was located on the middle and upper regions of a high mountain (the altitude of the mountain was greater than 4000 m.a.s.l.), such as those of Cg and Cg–N. Others were located on top of a large mountain area. The similar altitude and small-scale topographic features indicated that the three genera had close relationships and similar developmental histories. However, under the same mode, different slopes and aspects make the microclimate and vegetation community of each plot different, which provides the right conditions for the evolution of more diversity among *Chrysanthemum* and its allies in this study area.

### Correlation analysis based on plot data

There was at least one plot where AB was zero in the Cl, Cl–N, and Aq–N populations (Table S3), which indicated that the distribution range of these three populations was very narrow, as the maximum separation distance was 50 m. We found that the R was low, whereas the TLC was high and the SRC was low, in all plots. As mentioned above, Cg–N was a special population that was located on the northern slope with less light. However, the SRCs of the three plots were high, with values above 20%, and the highest value was 37%. This topography resulted in very low TLC, and the average TLC of the three plots was less than 3%, which reduced the shading of the shrub layer and herb layer from the perspective of the plant community and made up for the lack of light. In all six plots of *A. sericea*, the TLC was low, whereas the SLC, HLC, and SRC were high, and the average SRC was more than 20%. These stony southern slopes formed a relatively dry microclimate with a small area that provided the specific habitat and vegetation community, which is optimal for *A. sericea*. The composition of the tree layer displayed a certain consistency, often in the form of coniferous-evergreen, broad-leaved, mixed forest trees, mainly including *Pinus yunnanensis*, *Pinus armandii*, *Quercus aquifolioides*, *Quercus rehderiana*, and *Quercus spinosa*.

The AB of *Chrysanthemum* and its allies had significant positive correlations with SRC (*R* = 0.59, α = 0.05) and HLC (*R* = 0.67, α = 0.05) and was negatively related to TLC (*R* = −0.51, α = 0.05) and TS (*R* = −0.34, α = 0.05) (Fig. 3b). These findings indicated that all target species in this area were mainly distributed in open stony coniferous–broad-leaved, mixed forest margins, and they hardly grew in the dense woods. These marginal communities were similar to ecological islands, separated by other dense vegetation communities and scattered in small-scale areas formed by special stony topography.

## Discussion

### Factors affecting the particularity of distribution

The genus *Chrysanthemum* is generally distributed in the middle-low altitude and middle-low-latitude humid areas of the subtropical and temperate zones of Asia, and a few grow in the high-latitude frigid zones of Russia (Far East) and North America. For *C. indicum*, the distribution area is relatively wide, but there are few high-altitude populations^25^. *Ajania* is usually distributed in mid-latitude arid and semiarid areas, and the altitude is higher than that of *Chrysanthemum*, with the highest altitude being 5300 m.a.s.l. (*A. khartensis*). Ci was the highest population of *Chrysanthemum indicum* (3210 m.a.s.l.) reported in China, with an altitude higher than that of *Ajania* in this area. This may be mainly due to the relatively warm climate of the low latitudes. In addition, this area is located in the southernmost margin of the Hengduan Mountain Range, which blocks the cold current from the north. The warm and wet current from the south^59^ easily forms rain and fog at the top of the mountains, which makes the small area relatively warm and humid even in the high-altitude areas above 3000 m.a.s.l., allowing *C. indicum* to survive. In addition, these particular stony small areas with long light hours and a relatively dry microclimate allowed *Ajania* to exist in the low-latitude area with 1000 mm annual average precipitation. However, the differences in light hours caused by aspect and vegetation community led to the discrepant florescence of *Chrysanthemum* in this area. Even for the same species, the florescence of the Cg–N population on the northern aspect occurred earlier than that of the Cg population on the eastern aspect (Table 1).

However, there are many similar stony topographies with relatively low altitudes in this area, so why is there no distribution of *Chrysanthemum* and its allies? We found that their preferred habitats were highly dependent on the specialized vegetation community: alpine coniferous–broad-leaved mixed forest marginal communities, in which the conifers were mainly from *Pinus*, whereas the broad-leaved communities were usually alpine evergreen sclerophyllous *Quercus* and *Rhododendron*. We highlight that the development of these communities^60^ is of great significance to the distribution and evolution of *Chrysanthemum* and its allies, and this region has become an ecological island^61^ of refuge^28,62^ for these species in the current interglacial period.

### Migration path and divergence time estimation based on paleopalynology, paleobotany, and paleogeography

**Fig. 6:**
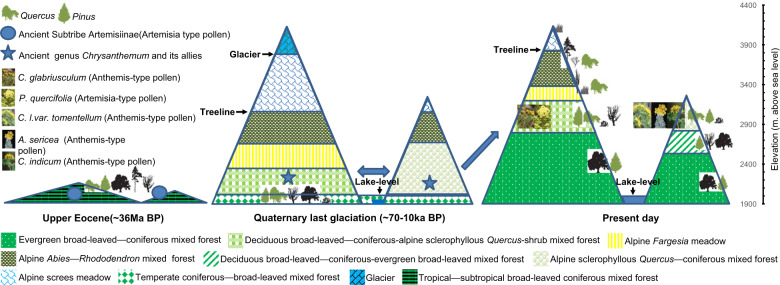
Paleoelevation and paleovegetation of the Upper Eocene (~36 Ma BP) and the last Quaternary glaciation (~70–10 ka BP) for two types of mountain ranges studied based on previous studies. The potential migration path and evolutionary history of *Chrysanthemum* and its allies was proposed based on comprehensive analysis of present-day (this study) and ancient topography and vegetation

Based on the data and paleopalynological, paleobotanical, and paleogeographical^63^ evidence, we reconstructed the ancient topography and vegetation^64^, ensuring that the estimations of the origin, divergence time^65,66^, and migration path of these species are as accurate as possible. This study area is the original location of “Tali Glaciation”^67–69^. A large amount of relevant research data provides strong support for exploring the change in climate and evolutionary history of vegetation in this area. According to the current altitude data, the mountains around Erhai Lake were divided into two types: those with an altitude of more than 3900 m.a.s.l. and those with an altitude of more than 3000 m.a.s.l. but less than 3300 m.a.s.l. We then reconstructed the paleoelevation and paleovegetation of the Upper Eocene (~36 Ma BP)^50,51,70,71^ and the last Quaternary glaciation (~70–10 Ka BP)^46,72,73^ for these two types of mountains (Fig. 6).

The ancestral range of Anthemideae Cass. was most likely in Central and South Africa during the Upper Eocene^74^, approximately 37–36 Ma BP. This study area had a paleoelevation of 1.3–2.6 km above sea level, which would most likely have been associated with a vegetation cover consisting of tropical-subtropical, deciduous, coniferous, broadleaf forests that were mainly composed of *Carya*, *Juglans*, *Castanea*, *Alnus*, *Polypodium*, *Pinus*, *Quercus*, and *Abies* (Fig. 6). The reconstructed mean annual air temperature (MAAT) had a value of 16.8–21.7 °C, warmer than the present-day MAAT (~6 °C). However, there was an ancient subtribe, Artemisiinae (Anthemideae), in Jianchuan based on Artemisiaepollenites (Artemisia-type pollen)^51^. Therefore, we speculated that the ancient Artemisiinae with Artemisia-type pollen was a relatively primitive vernacular Anthemideae group in this region and was the common ancestor of *Artemisia*, *Phaeostigma*, *Chrysanthemum*, and *Ajania*.

By the last Quaternary glaciation (~70–10 ka BP), the Qinghai–Tibetan Plateau and Hengduan Mountains had been uplifted, and the landform of this area was basically the same as it is now. Affected by glaciation, the climate had become cold and dry, the area above 3900 m.a.s.l. was covered by glaciers all year round, and the lake level had reduced. The climate in the low-altitude basin area was relatively warm and became that of a temperate coniferous–broad-leaved mixed forest dominated by *Pinus Cyclobalanopsis*, *Tsuga*, and *Quercus*^46^ (Fig. 6), which was similar to the community that *Chrysanthemum* and its allies is dependent on today. Despite the lack of pollen fossils in this stratum, we inferred that *Chrysanthemum* and *Ajania* or their common ancestor already existed in this area based on their present communities. However, the populations were not large, scattered in the open or located in the margins of forests, but they started lateral radiation and migration in the low-altitude and middle-altitude (1900–2500 m.a.s.l.) areas (Fig. 6). In the Holocene (interglacial stage), the climate had become humid and warm, with dense vegetation in low-altitude and medium-altitude areas, forming the current evergreen broad-leaved coniferous mixed forests mainly composed of *Pinus*, *Alnus*, and *Castanopsis*. Owing to competition between populations, the habitat of *Chrysanthemum* and its allies has become compressed, and the populations have been forced to migrate to high-altitude areas with the coniferous–broad-leaved mixed forest marginal community, thus forming the high-altitude scattered distribution pattern seen at present. The Quaternary has experienced several glacial-interglacial cycles^75^, but we could not reconstruct the vegetation of the whole Quaternary owing to the lack of data. However, notably, the geodiversity caused by the uplift of the Hengduan Mountains created a small-scale^76^ alpine topography with diverse microclimates, which provided a refuge for *Chrysanthemum* and its allies during the interglacial stage and drove the divergence and diversification of their populations. The pollen fossils of *Chrysanthemum* and *Ajania* have been found only in the strata of the late Quaternary (~20 Ka Bp) to date. Hence, we estimated that the divergence of the two genera occurred along with the uplift of the Hengduan Mountains and Quaternary glacial-interglacial cycles, from approximately the late Miocene to the late Pleistocene (8–0.1 Ma).

### Evolutionary relationships among *Chrysanthemum*, *Ajania*, and *Phaeostigma*

Based on the brownish style branches, erect corolla lobes, and microechinate pollen, Muldeshev^12,13^ separated *Phaeostigma* from *Ajania*. However, there were also brownish style branches in *A. sericea*, whereas the browning degree was relatively lower than that in *P. quercifolium*. Two different pollen exine ornamentations have been confirmed for the tribe Anthemideae, namely, the Anthemis-type (echinate, with medium to long spines) and the Artemisia-type (microechinate, with short spinules)^77,78^. The former was more likely to be related to entomophily, while the latter was more likely to be related to anemophily^78,79^. *Chrysanthemum* and most *Ajania* species Anthemis-type species, while *Phaeostigma* species were Artemisia-type species^15,17,78^, and there was no relevant research on *A. sericea*. In our study, although pollen morphology was not involved, *P. quercifolium* was more likely related to anemophily, and the other four species, including *A. sericea*, were more likely related to entomophily based on the pollinators recorded through our long-term field work (Fig. S3), which strongly supported the previous research hypothesis that *Phaeostigma* and *Ajania* were split, based on ecological evidence. In addition, our results on the morphological characteristics also supported this hypothesis, with differences in the qualitative traits of *P. quercifolium* from those reported in previous studies, such as a subshrub structure, the presence of small scars on the stem, lateral expansion of the petiole base, yellow-white tubular florets, erect corolla lobes, the absence of emergent rhizomes, and annular lobules at the petiole. Last, our cluster analysis based on SRAP markers provided genetic evidence for this hypothesis: the genetic relationship between *Phaeostigma* and *Chrysanthemum* was relatively distant, and the former formed an independent cluster that was separate from *Chrysanthemum* and *Ajania* (Fig. 4).

For *Chrysanthemum* and *Ajania*, despite the observation that the populations of *Ajania* were separated from those of *Chrysanthemum*, based on our PCA results, we suggest that the two genera were very closely related based on the obvious smooth transition between states for marginal female florets between *Chrysanthemum* (ray florets) and *Ajania* (tubular florets) discovered in the Cg–N population (*C. glabriusculum*) and the fact that they use the same entomophily pollination system. This assumption is also supported by previous molecular biology results^15,17^, and our dendrogram showed that the genetic distance between *Chrysanthemum* and *Ajania* was closer than that between *Chrysanthemum* and *Phaeostigma* (Fig. 4).

However, there is a key problem: what were the ancestral characteristics of the marginal female florets, the tubular florets, or ray florets? Previous studies have shown that Artemisia groups (tubular florets) were considered to have evolved secondarily by loss of ray florets^19,78,80^. However, the discovery of a number of fossil pollen (with short spinules) grains preserved in dinosaur-bearing deposits from the Late Cretaceous (~76–66 Ma) in Antarctica drastically pushes back the timing of assumed origin of Asteraceae^65^. Therefore, at the family and angiosperm levels, we speculate that the groups that were likely related to anemophily with short spinule pollens were the more ancient and primitive ancestors. Hence, tubular florets corresponding to anemophily may be the ancestral characteristics of marginal female florets. The results of our PCA and qualitative trait analysis indicated that *Chrysanthemum* had a more significant inflorescence to attract pollinators; in contrast, *P. quercifolium* was unattractive, with erect whitish corollas, and *A. sericea* was in the middle of the two. *P. quercifolium* may be a relic species of Artemisiinae that benefited from the diverse topographies and climate of the Hengduan Mountains, which was a refuge for this species, as it survived through several global climate changes in the Late Tertiary and Quaternary.

Owing to the limitations of species samples, in this study, we were unable to perform a comprehensive analysis of the evolution of the three genera. However, we highlight that *Chrysanthemum* and its related genera were highly dependent on small-scale special stony topographies and specialized communities in the study area and that the northern aspect (16.28), with less light, may be the key factor for the elongation and evolution of marginal female florets. The uplift of the Hengduan Mountains played a key role in the evolution, divergence, and survival of *Chrysanthemum* and its allies.

## Supplementary information

Table S1

Table S2

Table S3

Tabla S4

Table S5

Table S6

Fig.S1(a)

Fig.S1(b)

Fig.S1(c)

Fig.S1(d)

Fig.S2

Fig.S3

Fig.S4

Fig. S5
