# Supplementary material for: Small-scale alpine topography at low latitudes and high altitudes: refuge areas of the genus Chrysanthemum and its allies
Source: Hortic Res. 2020 Nov 1;7:184. doi: 10.1038/s41438-020-00407-9 (PMC7603505; doi:10.1038/s41438-020-00407-9)
Supplement: Supplementary file 1 — Table S1 [file 41438_2020_407_MOESM1_ESM.docx]

**Table S1** Sequence of forward and reverse SRAP primers used in this study

| Forward primer | Primer sequence（5’ to 3’） | Reverse primer | Primer sequence（5’ to 3’） |
| --- | --- | --- | --- |
| Me10 | TGAGTCCAAACCGGATG | Em1 | GACTGCGTACGAATTAAT |
| Me13 | TGAGTCCAAACCGGTAA | Em2 | GACTGCGTACGAATTTGC |
| Me15 | TGAGTCCAAACCGGTGC | Em4 | GACTGCGTACGAATTTGA |
| Me17 | AGCGAGCAAGCCGGTGG | Em5 | GACTGCGTACGAATTAAC |
| Me19 | CAAATGTGAACCGGATA | Em6 | GACTGCGTACGAATTGCA |
| Me20 | GAGTATCAACCCGGATT | Em7 | GACTGCGTACGAATTATG |
| Me21 | GTACATAGAACCGGAGT | Em8 | GACTGCGTACGAATTAGC |
| Me22 | TACGACGAATCCGGACT | Em9 | GACTGCGTACGAATTACG |
| Me23 | CACAGTCATGCCGGAAT | Em10 | GACTGCGTACGAATTTAG |
| Me24 | GACCAGTAAACCGGATG | Em14 | GACTGCGTACGAATTCAG |
|  |  | Em15 | GACTGCGTACGAATTCTG |
|  |  | Em16 | GACTGCGTACGAATTCGG |
