# Supplementary material for: Small-scale alpine topography at low latitudes and high altitudes: refuge areas of the genus Chrysanthemum and its allies
Source: Hortic Res. 2020 Nov 1;7:184. doi: 10.1038/s41438-020-00407-9 (PMC7603505; doi:10.1038/s41438-020-00407-9)
Supplement: Supplementary file 2 — Table S2 [file 41438_2020_407_MOESM2_ESM.docx]

| Primer combination | Number of bands scored | Polymorphic bands | Ratio of polymorphism | Primer combination | Number of bands scored | Polymorphic bands | Ratio of polymorphism |
| --- | --- | --- | --- | --- | --- | --- | --- |
| M13E4 | 14 | 13 | 92.86 | M22E1 | 7 | 7 | 100.00 |
| M17E9 | 16 | 16 | 100.00 | M22E4 | 7 | 7 | 100.00 |
| M19E15 | 13 | 11 | 84.62 | M22E14 | 10 | 10 | 100.00 |
| M22E7 | 8 | 8 | 100.00 | M22E15 | 10 | 10 | 100.00 |
| M22E8 | 12 | 12 | 100.00 | M23E5 | 13 | 13 | 100.00 |
| M22E9 | 16 | 16 | 100.00 | M23E6 | 12 | 11 | 91.67 |
| M20E4 | 14 | 12 | 85.71 | M22E5 | 15 | 15 | 100.00 |
| M20E5 | 12 | 12 | 100.00 | M24E15 | 7 | 7 | 100.00 |
| M20E10 | 12 | 12 | 100.00 | M24E16 | 12 | 12 | 100.00 |
| M21E1 | 14 | 13 | 92.86 | M10E8 | 11 | 11 | 100.00 |
| M21E2 | 14 | 14 | 100.00 | M15E7 | 12 | 12 | 100.00 |
| M21E4 | 8 | 8 | 100.00 | Total | 276 | 269 | - |
| M21E6 | 7 | 7 | 100.00 | Average | 11.50 | 11.20 | 97.46.1% |

**Table S2** Total and polymorphic bands of each SRAP primer
