# Supplementary material for: Small-scale alpine topography at low latitudes and high altitudes: refuge areas of the genus Chrysanthemum and its allies
Source: Hortic Res. 2020 Nov 1;7:184. doi: 10.1038/s41438-020-00407-9 (PMC7603505; doi:10.1038/s41438-020-00407-9)
Supplement: Supplementary file 3 — Table S3 [file 41438_2020_407_MOESM3_ESM.docx]

**Table S3** The abundance(AB),species richness(R),trees taxon and coverage of each layer by plots for 9 populations

| Population | TS | SS | HS | R | TLC(%) | SLC(%) | HLC(%) | SRC(%) | AB | Trees taxon |
| --- | --- | --- | --- | --- | --- | --- | --- | --- | --- | --- |
| Cg1 | 4 | 8 | 26 | 38 | 28 | 65 | 39 | 8 | 8 | *Populus davidiana*(*Po.davidiana*);  *Pinus yunnanensis*(*P.yunnanensis*);  *Pinus armandii*(*P.armandii*);  *Sorbus rehderiana*(*S.rehderiana*) |
| Cg2 | 2 | 9 | 28 | 39 | 24 | 63 | 45 | 12 | 11 | *Po.davidiana; P.yunnanensis* |
| Cg3 | 2 | 6 | 24 | 32 | 17 | 48 | 41 | 3 | 5 | *P.armandii; S.rehderiana* |
| Cg-N1 | 0 | 13 | 34 | 45 | 0 | 78 | 52 | 23 | 4 |  |
| Cg-N2 | 2 | 15 | 31 | 48 | 7 | 73 | 48 | 31 | 6 | *Illicium simonsii; S.rehderiana* |
| Cg-N3 | 0 | 9 | 29 | 38 | 0 | 41 | 42 | 37 | 11 |  |
| Ci1 | 1 | 8 | 17 | 28 | 8 | 62 | 45 | 15 | 13 | *Q.rehderiana* |
| Ci2 | 3 | 9 | 12 | 24 | 27 | 5 | 33 | 22 | 10 | *Q.rehderiana; Q.aquifolioides;*  *Quercus spinosa*(*Q.spinosa*) |
| Ci3 | 4 | 5 | 15 | 24 | 78 | 18 | 22 | 0 | 0 | *Q.rehderiana; P.armandii;*  *P.yunnanensis; Rhododendron yunnanense* |
| Cl1 | 1 | 1 | 30 | 32 | 12 | 7 | 85 | 23 | 27 | *Quercus rehderiana*(*Q.rehderiana*) |
| Cl2 | 1 | 2 | 18 | 21 | 87 | 2 | 12 | 0 | 2 | *Q.rehderiana* |
| Cl3 | 3 | 3 | 9 | 15 | 96 | 5 | 11 | 0 | 0 | *Q.rehderiana; P.armandii*  *R.cuneatum* |
| Cl-N1 | 2 | 3 | 23 | 28 | 92 | 6 | 19 | 11 | 0 | *P.armandii; Quercus aquifolioides*  (*Q.aquifolioides*) |
| Cl-N2 | 3 | 1 | 27 | 31 | 78 | 9 | 23 | 16 | 5 | *P.armandii; Q.aquifolioides P.yunnanensis* |
| Cl-N3 | 4 | 3 | 36 | 43 | 67 | 8 | 49 | 22 | 8 | *P.armandii; Q.aquifolioides;*  *P.yunnanensis; Rhododendron cuneatum*  (*R.cuneatum*) |
| As1 | 2 | 5 | 15 | 22 | 16 | 21 | 53 | 32 | 26 | *Q.rehderiana; P.yunnanensis* |
| As2 | 1 | 4 | 15 | 20 | 8 | 37 | 59 | 12 | 12 | *Q.rehderiana* |
| As3 | 1 | 2 | 19 | 22 | 36 | 37 | 49 | 19 | 21 | *P.yunnanensis* |
| As-N1 | 2 | 3 | 21 | 26 | 21 | 47 | 31 | 7 | 3 | *Q.rehderiana; P.yunnanensis* |
| As-N2 | 1 | 4 | 16 | 21 | 4 | 39 | 25 | 39 | 15 | *Q.rehderiana* |
| As-N3 | 1 | 4 | 17 | 22 | 15 | 48 | 21 | 23 | 9 | *Q.rehderiana* |
| Pq1 | 3 | 7 | 24 | 32 | 62 | 20 | 46 | 33 | 12 | *Q.rehderiana; P.armandii;*  *P.yunnanensis* |
| Pq2 | 4 | 6 | 23 | 33 | 6 | 23 | 37 | 16 | 7 | *Q.rehderiana; P.yunnanensis;*  *P.armandii; Q.aquifolioides* |
| Pq3 | 4 | 4 | 27 | 35 | 53 | 23 | 40 | 24 | 11 | *Q.rehderiana; P.yunnanensis;*  *P.armandii; Q.aquifolioides* |
| Pq-N1 | 5 | 8 | 22 | 35 | 87 | 21 | 33 | 0 | 0 | *Q.rehderiana; P.yunnanensis;*  *P.armandii; Viburnum cylindricum;*  *Rhamnus virgata* |
| Pq-N2 | 3 | 12 | 31 | 46 | 42 | 39 | 66 | 12 | 13 | *Q.rehderiana; P.yunnanensis;*  *Rhododendron traillianum* |
| Pq-N3 | 2 | 9 | 27 | 38 | 35 | 58 | 59 | 17 | 7 | *Q.rehderiana; P.yunnanensis* |
