# Supplementary material for: Small-scale alpine topography at low latitudes and high altitudes: refuge areas of the genus Chrysanthemum and its allies
Source: Hortic Res. 2020 Nov 1;7:184. doi: 10.1038/s41438-020-00407-9 (PMC7603505; doi:10.1038/s41438-020-00407-9)
Supplement: Supplementary file 4 — Tabla S4 [file 41438_2020_407_MOESM4_ESM.docx]

**Table S4** The values of altitude,aspect,slope and topographic position index of 9 populations

| Population | Altitude | Aspect | Slope | Topographic position Index |
| --- | --- | --- | --- | --- |
| Cg | 3113 | 67.75 | 28.80 | 3 |
| Cg-N | 2754 | 16.27 | 32.66 | 2 |
| Ci | 3177 | 126.20 | 26.03 | 3 |
| Cl | 3029 | 176.64 | 21.93 | 3 |
| Cl-N | 3177 | 151.83 | 24.14 | 3 |
| As | 2914 | 193.06 | 15.90 | 3 |
| As-N | 2871 | 183.52 | 31.74 | 3 |
| Pq | 3116 | 226.49 | 35.63 | 2 |
| Pq-N | 3218 | 261.39 | 14.58 | 3 |
