# Supplementary material for: Small-scale alpine topography at low latitudes and high altitudes: refuge areas of the genus Chrysanthemum and its allies
Source: Hortic Res. 2020 Nov 1;7:184. doi: 10.1038/s41438-020-00407-9 (PMC7603505; doi:10.1038/s41438-020-00407-9)
Supplement: Supplementary file 5 — Table S5 [file 41438_2020_407_MOESM5_ESM.docx]

**Table S5** Morphology code, diversity Index (*H’*) and coefficient of variation (CV) for 9 populations

| № | Morphology | Code | Cg | Cg-N | Ci | CL | CL-N | As | As-N | Pq | Pq-N | Mean | SD | CV | *H′* |
| --- | --- | --- | --- | --- | --- | --- | --- | --- | --- | --- | --- | --- | --- | --- | --- |
| 1 | Lignified Degree | Herbs 0/Subshrubs 1 | 0 | 0 | 0 | 0 | 0 | 0 | 0 | 1 | 1 | 0.22 | 0.44 | 198.43% | 0.53 |
| 2 | Stem | Erect 0/Diffuse1 | 0 | 1 | 0 | 0 | 1 | 0 | 0 | 0 | 0 | 0.22 | 0.44 | 198.43% | 0.63 |
| 3 | Lateral branch | None 0/ Exist 1 | 1 | 1 | 1 | 1 | 1 | 0 | 0 | 1 | 1 | 0.78 | 0.44 | 56.69% | 0.53 |
| 4 | Procumbent rhizome | None 0/Exist 1 | 1 | 1 | 1 | 1 | 1 | 0 | 0 | 0 | 0 | 0.56 | 0.53 | 94.87% | 0.69 |
| 5 | Colors of basal stem | Dark Gray 0/Brown 1/gray-white 2 | 1 | 1 | 1 | 1 | 1 | 2 | 2 | 0 | 0 | 1.00 | 0.71 | 70.71% | 0.99 |
| 6 | Colors of Middel stem | Green 0/Purple 1/Grayish White 2 | 1 | 1 | 1 | 0 | 0 | 2 | 2 | 1 | 1 | 1.00 | 0.71 | 70.71% | 0.99 |
| 7 | Color of anthocaulus | Green 0//Grayish White 1 | 1 | 1 | 1 | 0 | 0 | 1 | 1 | 1 | 1 | 0.78 | 0.44 | 56.69% | 0.53 |
| 8 | Longitudinal ridge of Mid-upper stem | None 0/Shallow 1/Deep 2 | 1 | 1 | 1 | 0 | 0 | 1 | 1 | 2 | 1 | 0.89 | 0.60 | 67.60% | 0.84 |
| 9 | Indumentum of Mid-upper stem | Puberulent 0/Villous 1/Powdery-sericeous 2/Densely and thickly Tomentose-Sericeous 3 | 0 | 0 | 0 | 1 | 1 | 3 | 3 | 2 | 2 | 1.33 | 1.22 | 91.86% | 1.37 |
| 10 | Indumentum of Basal stem | Glabrous 0/Pilose 1/Tomentose-Sericeous 2 | 0 | 1 | 0 | 1 | 1 | 2 | 2 | 0 | 0 | 0.78 | 0.83 | 107.14% | 1.06 |
| 11 | Emergent rhizome | None 0/ Exist 1 | 1 | 1 | 1 | 1 | 1 | 1 | 1 | 0 | 0 | 0.78 | 0.44 | 56.69% | 0.53 |
| 12 | Leaf texture | Chartaceous 0/Rigid-chartaceous 1 | 0 | 0 | 0 | 0 | 0 | 0 | 0 | 1 | 1 | 0.22 | 0.44 | 198.43% | 0.53 |
| 13 | Same/Similar color on both surfaces of leaf | No 0/Yes 1 | 0 | 0 | 0 | 0 | 1 | 1 | 1 | 0 | 0 | 0.33 | 0.50 | 150.00% | 0.63 |
| 14 | Adaxially leaf Colors | Green 0/Gray-white 1 | 0 | 0 | 0 | 0 | 0 | 1 | 1 | 0 | 0 | 0.22 | 0.44 | 198.43% | 0.53 |
| 15 | Abaxially leaf Colors | Green 0/Gray-white 1 | 1 | 1 | 1 | 1 | 0 | 1 | 1 | 1 | 1 | 0.89 | 0.33 | 37.50% | 0.35 |
| 16 | Middle stem leaf Shapes | Ovate 0/ Elliptic-Ovate 1/Lanceolate 2/ Flabelliform3/Narrowly Elliptic 4 | 0 | 0 | 0 | 1 | 1 | 3 | 3 | 2 | 4 | 1.56 | 1.51 | 97.02% | 1.52 |
| 17 | Middle stem leaf Divisions | Margin coarsely Dentate 0/2-Pinnatisecta 1/3-Pinnatisecta 2/3-Subtripalmate-Pinnatisect 3 | 1 | 1 | 1 | 1 | 1 | 3 | 2 | 0 | 0 | 1.11 | 0.93 | 83.52% | 1.14 |
| 18 | Degree of primary division | None 0/ Deeply-divided 1/Totally-divided2 | 2 | 2 | 2 | 2 | 1 | 2 | 2 | 0 | 0 | 1.44 | 0.88 | 61.06% | 0.84 |
| 19 | Primary lateral segments or teeth | 2-paired 0/3-paired 1 /4-paired 2 | 0 | 1 | 0 | 0 | 0 | 0 | 0 | 2 | 1 | 0.44 | 0.73 | 163.46% | 0.84 |
| 20 | Ultimate segments shapes | Obliquely triangular 0/Narrowly elliptic 1 /Lanceolate 2/Broadly elliptic 3 | 0 | 0 | 2 | 3 | 3 | 1 | 1 | 0 | 0 | 1.11 | 1.27 | 114.24% | 1.27 |
| 21 | Lobe margins awn-liked dentate | None 0/ Exist 1 | 1 | 1 | 1 | 1 | 1 | 0 | 0 | 0 | 0 | 0.56 | 0.53 | 94.87% | 0.69 |
| 22 | Upper stem leaf shapes | Ovate 0/ Eliptic-ovate 1/Lanceolate 2/Suborbicular 3/Narrowly elliptic 4 | 1 | 0 | 0 | 4 | 4 | 3 | 3 | 2 | 2 | 2.11 | 1.54 | 72.79% | 1.58 |
| 23 | Upper stem leaf divisions | Entire 0/Margin coarsely dentate 1/Inconspicuously-divided 2/Halfly-divided 3/Subtripalmate-pinnatisect 4 | 3 | 2 | 3 | 1 | 1 | 4 | 4 | 1 | 0 | 2.11 | 1.45 | 68.82% | 1.52 |
| 24 | Adaxially leaf indumentum | Glabrous 0/Sparsely pubescent 1/Sparsely villous 2/Densely and thickly tomentose 3 | 0 | 0 | 0 | 1 | 2 | 3 | 3 | 0 | 0 | 1.00 | 1.32 | 132.29% | 1.14 |
| 25 | Abaxially leaf indumentum | Densely and thickly appressed pubescent 0/Densely villous 1/Densely and thickly sericeous 2/Densely and thickly sericeous-tomentose 3 | 0 | 0 | 0 | 1 | 1 | 3 | 3 | 2 | 2 | 1.33 | 1.22 | 91.86% | 1.37 |
| 26 | Annular lobules at petiole base | None 0/ Exist 1 | 1 | 1 | 1 | 1 | 1 | 1 | 1 | 0 | 0 | 0.78 | 0.44 | 56.69% | 0.53 |
| 27 | Lateral expansion of petiole base | None 0/ Exist 1 | 0 | 0 | 0 | 0 | 0 | 0 | 0 | 1 | 1 | 0.22 | 0.44 | 198.43% | 0.53 |
| 28 | Cicatricle | None 0/ Exist 1 | 0 | 0 | 0 | 0 | 0 | 0 | 0 | 1 | 1 | 0.22 | 0.44 | 198.43% | 0.53 |
| 29 | Leaves arranged on stem | Sparsely 0/Medially 1/Densely 2 | 1 | 1 | 1 | 1 | 0 | 2 | 2 | 1 | 1 | 1.11 | 0.60 | 54.08% | 0.84 |
| 30 | Synflorescence flat-topped panicle | Sparse 0/Medium 1/Dense 2 | 1 | 1 | 1 | 0 | 0 | 2 | 2 | 2 | 2 | 1.22 | 0.83 | 68.18% | 1.06 |
| 31 | Involucres | Campanulate 0/Cup-shaped 1 | 0 | 0 | 0 | 1 | 1 | 0 | 0 | 0 | 0 | 0.22 | 0.44 | 198.43% | 0.53 |
| 32 | Shapes of outer phyllaries | Linear 0 / Ovate-lanceolate1 / Triangular-lanceolate 2 | 2 | 2 | 2 | 2 | 2 | 0 | 0 | 1 | 1 | 1.33 | 0.87 | 64.95% | 0.99 |
| 33 | Indumentum of outer phyllaries | Glabrous 0/Sparsely sericeous 1/Sparsely pubescent 2/Densely and thickly sericeous-tomentos 3 | 0 | 0 | 0 | 2 | 2 | 3 | 3 | 1 | 1 | 1.33 | 1.22 | 91.86% | 1.37 |
| 34 | Colors of phyllaries scarious margin | White-yellow 0/Yellow-Brown 1/Pale brown 2/Dark brown 3 | 3 | 2 | 2 | 2 | 2 | 0 | 2 | 1 | 1 | 1.67 | 0.87 | 51.96% | 1.14 |
| 35 | Receptacle | Hemispherical-Conical 0/Conical 1 | 1 | 1 | 1 | 0 | 0 | 1 | 1 | 1 | 1 | 0.78 | 0.44 | 56.69% | 0.53 |
| 36 | Sessile glands | None 0/ Exist 1 | 1 | 1 | 1 | 1 | 1 | 1 | 1 | 1 | 1 | 1.00 | 0.00 | 0.00% | 0.00 |
| 37 | Ray florets | (0) 0/(3-8) 1/(9-20) 2 | 1 | 2 | 1 | 2 | 2 | 0 | 0 | 0 | 0 | 0.89 | 0.93 | 104.40% | 1.06 |
| 38 | Ray florets lamina apex | None 0/Entire 1/2- or 3-denticulate 2 | 2 | 1 | 2 | 1 | 1 | 0 | 0 | 0 | 0 | 0.78 | 0.83 | 107.14% | 1.06 |
| 39 | Marginal female tubular florets number | None 0/(3-8)1/(9-15) 2/ | 0 | 0 | 0 | 0 | 0 | 1 | 1 | 2 | 2 | 0.67 | 0.87 | 129.90% | 0.99 |
| 40 | Marginal female tubular florets apex | None 0/4-denticulate 1/5-denticulate 2 | 0 | 0 | 0 | 0 | 0 | 2 | 1 | 1 | 1 | 0.56 | 0.73 | 130.77% | 0.93 |
| 41 | Tubular florets number | (20-35) 0/(35-50) 1 | 0 | 1 | 0 | 1 | 1 | 1 | 1 | 0 | 0 | 0.56 | 0.53 | 94.87% | 0.69 |
| 42 | Colors of tubular florets | Yellow 0/Yellow-white1 | 0 | 0 | 0 | 0 | 0 | 0 | 0 | 1 | 1 | 0.22 | 0.44 | 198.43% | 0.53 |
| 43 | Corolla lobes | Outward-curved 0/Erect 1 | 0 | 0 | 0 | 0 | 0 | 0 | 0 | 1 | 1 | 0.22 | 0.44 | 198.43% | 0.53 |
| 44 | Achenes | 4-ribbed 0/5-ribbed 1/6-ribbed 2 | 1 | 1 | 1 | 1 | 1 | 0 | 0 | 2 | 2 | 1.00 | 0.71 | 70.71% | 0.99 |
