# Supplementary material for: Small-scale alpine topography at low latitudes and high altitudes: refuge areas of the genus Chrysanthemum and its allies
Source: Hortic Res. 2020 Nov 1;7:184. doi: 10.1038/s41438-020-00407-9 (PMC7603505; doi:10.1038/s41438-020-00407-9)
Supplement: Supplementary file 6 — Table S6 [file 41438_2020_407_MOESM6_ESM.docx]

**Table S6** Quantitative traits (Mean + SD) and independent-sample t test between two populations of same species for 9 populations(*, *p*＜0.05).

| Population | PH（cm) | SD (mm) | MLL (cm) | MLW (cm) | PL (cm) | CCD(cm) | CD(mm) | RFL(mm) | IL(mm) | FTL(mm) | TL(mm) | AL(mm) |
| --- | --- | --- | --- | --- | --- | --- | --- | --- | --- | --- | --- | --- |
| Cg | 54.42±4.74 | 5.71±0.55 | 5.28±0.39 | 2.80±0.39 | 2.04±0.32 | 9.23±1.56 | 8.47±0.70 | 1.70±0.34 | 5.80±0.49 | — | 2.32±.0.17 | 1.65±0.13 |
| Cg-N | 69.96±9.22 | 5.85±0.71 | 5.89±0.60 | 2.94±0.47 | 2.19±0.41 | 11.02±1.61 | 9.77±1.30 | 2.41±0.71 | 5.86±0.49 | — | 2.60±0.18 | 1.62±0.13 |
| Ci | 52.95±6.78 | 5.21±0.46 | 4.96±0.50 | 2.45±0.46 | 1.62±0.35 | 8.34±0.95 | 12.56±1.77 | 4.07±0.67 | 5.24±0.35 | — | 2.29±0.21 | 1.47±0.12 |
| Cl | 41.94±5.62 | 4.83±0.48 | 1.91±0.35 | 5.03±0.53 | 1.36±0.13 | 11.18±2.04 | 14.48±0.92 | 5.57±1.00 | 4.93±0.34 | — | 2.84±0.23 | 1.41±0.10 |
| Cl-N | 51.66±5.73 | 5.00±0.56 | 1.95±0.32 | 5.07±0.42 | 1.58±0.33 | 12.2±1.43 | 15.08±1.03 | 5.09±0.71 | 5.00±0.31 | — | 3.06±0.25 | 1.45±0.10 |
| As | 39.40±6.34 | 3.76±0.62 | 1.79±0.24 | 0.90±0.23 | 0.23±0.07 | 5.90±1.04 | 4.38±0.38 | — | 3.38±0.39 | 1.48±0.22 | 1.89±0.22 | 0.95±0.16 |
| As-N | 36.80±4.58 | 3.18±0.29 | 1.53±0.12 | 0.88±0.21 | 0.19±0.06 | 5.45±1.07 | 4.32±0.51 | — | 3.06±0.32 | 1.37±0.08 | 1.90±0.15 | 0.93±0.20 |
| Aq | 85.69±12.08 | 7.42±0.72 | 2.52±0.38 | 7.75±0.57 | 0.50±0.15 | 8.24±0.71 | 5.29±0.54 | — | 4.88±0.34 | 2.25±0.24 | 3.12±0.19 | 1.50±0.16 |
| Aq-N | 73.46±11.04 | 6.87±0.73 | 3.30±1.20 | 6.56±1.30 | 0.50±0.08 | 7.93±0.94 | 5.26±0.47 | — | 4.78±0.42 | 2.17±0.23 | 3.01±0.20 | 1.44±0.13 |
|  | t *P* | t *P* | t *P* | t *P* | t *P* | t *P* | t *P* | t  *P* | t *P* | t *P* | t *P* | t *p* |
| Cg vs Cg-N | -4.74 0.000* | -0.49 0.630 | -2.48 0.023 | -0.72 0.479 | -0.92 0.372 | -2.52 0.021* | -2.79 0.015* | -2.85 0.014* | -0.27 0.788 | — — | -3.63 0.002* | 0.52 0.610 |
| Cl vs Cl-N | -3.83 0.001* | -0.73 0.743 | -0.19 0.853 | -0.27 0.793 | -1.97 0.072 | -1.30 0.213 | -1.39 0.186 | 1.24 0.234 | -0.48 0.636 | — — | -2.02 0.059 | -0.91 0.375 |
| As vs As-N | 1.05 0.309 | 2.68 0.019* | 0.20 0.842 | 3.11 0.008* | 1.43 0.169 | 0.96 0.352 | 0.30 0.768 | — — | 2.00 0.061 | 1.48 0.166 | -0.12 0.906 | 0.25 0.807 |
| Aq vs Aq-N | 2.36 0.030* | 1.70 0.107 | 2.65 0.021* | -1.95 0.077 | 0.00 1.000 | 0.83 0.416 | 1.33 0.896 | — — | 0.58 0.568 | 0.76 0.455 | 1.26 0.223 | 0.94 0.358 |
