# Supplementary figures and images for: Small-scale alpine topography at low latitudes and high altitudes: refuge areas of the genus Chrysanthemum and its allies
Source: Hortic Res. 2020 Nov 1;7:184. doi: 10.1038/s41438-020-00407-9 (PMC7603505; doi:10.1038/s41438-020-00407-9)

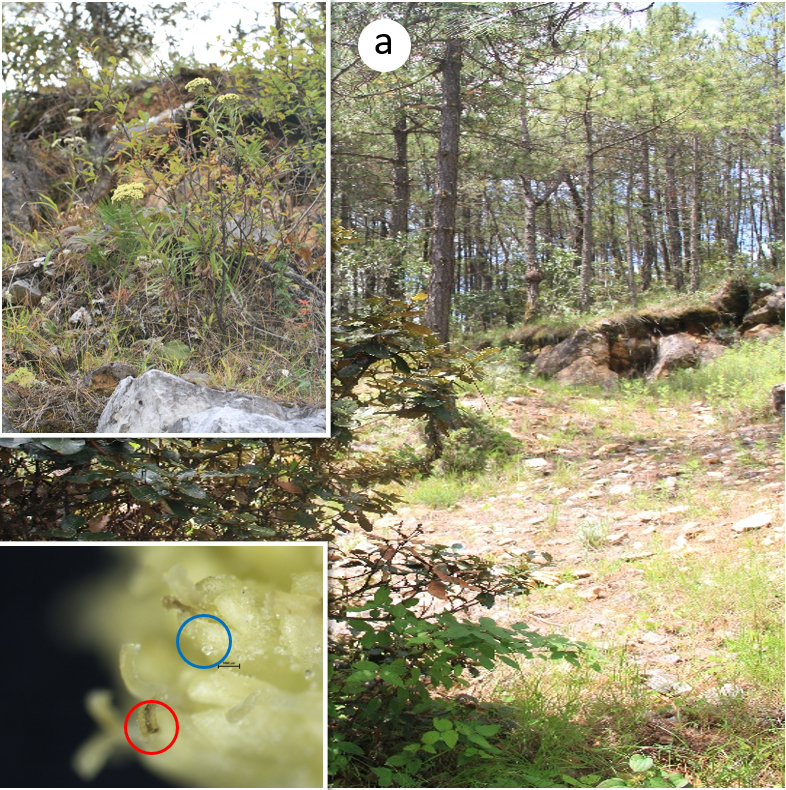

Supplement: Supplementary file 7 — Fig.S1(a) [file 41438_2020_407_MOESM7_ESM.jpg]

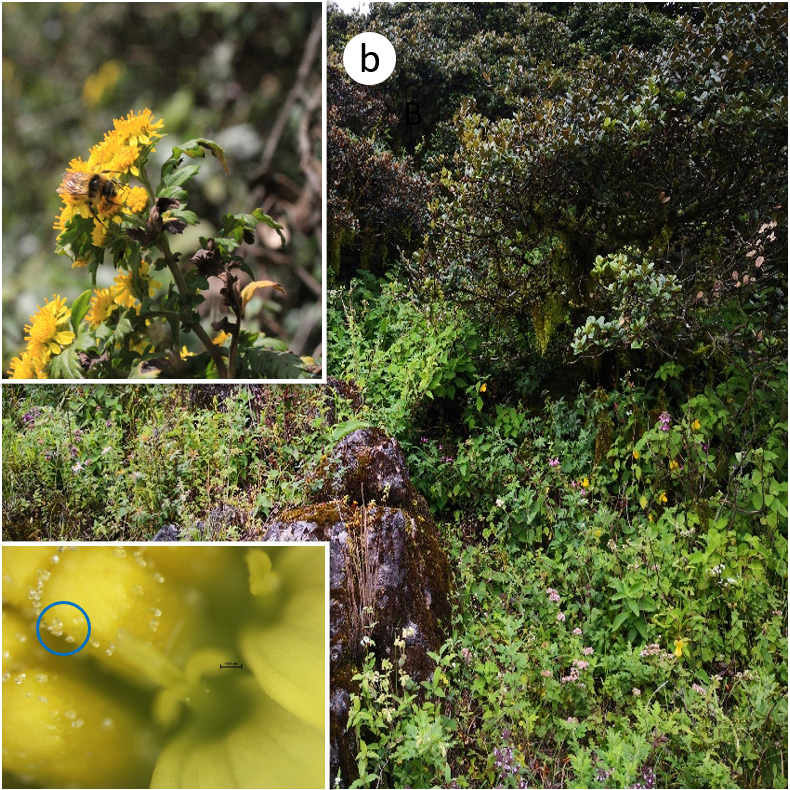

Supplement: Supplementary file 8 — Fig.S1(b) [file 41438_2020_407_MOESM8_ESM.jpg]

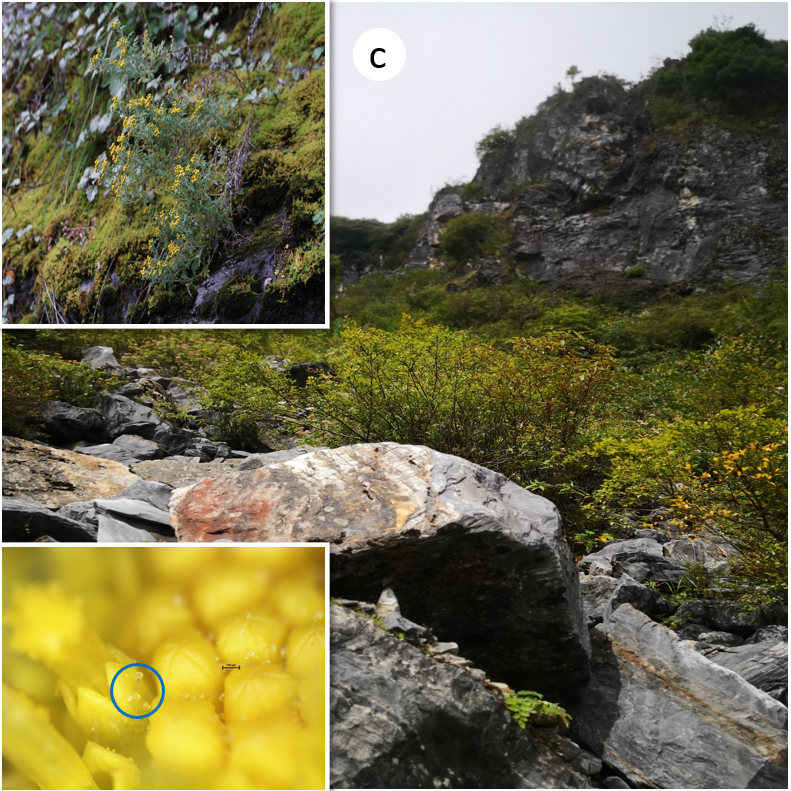

Supplement: Supplementary file 9 — Fig.S1(c) [file 41438_2020_407_MOESM9_ESM.jpg]

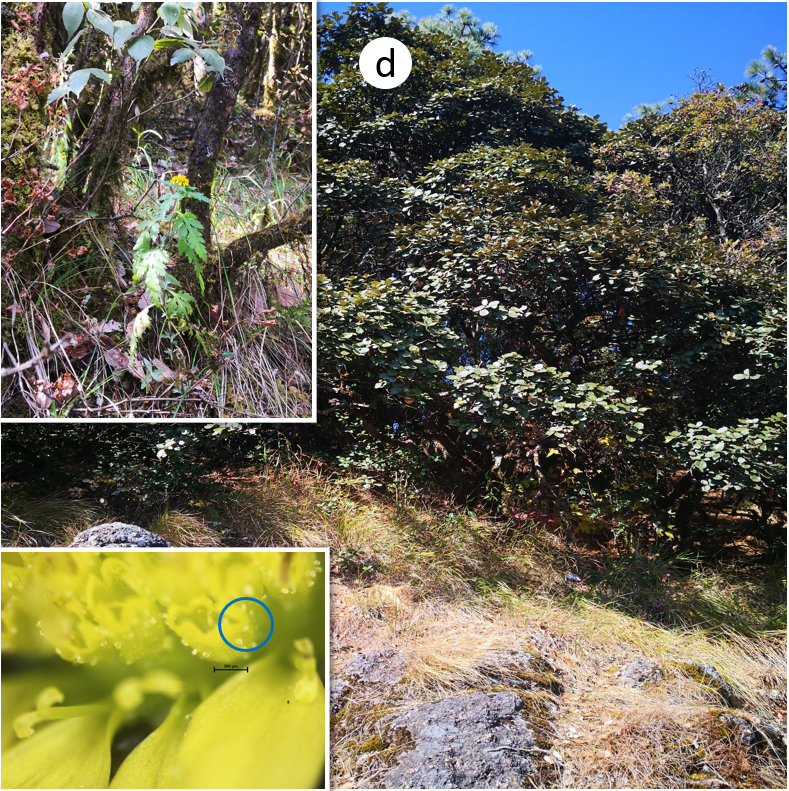

Supplement: Supplementary file 10 — Fig.S1(d) [file 41438_2020_407_MOESM10_ESM.jpg]

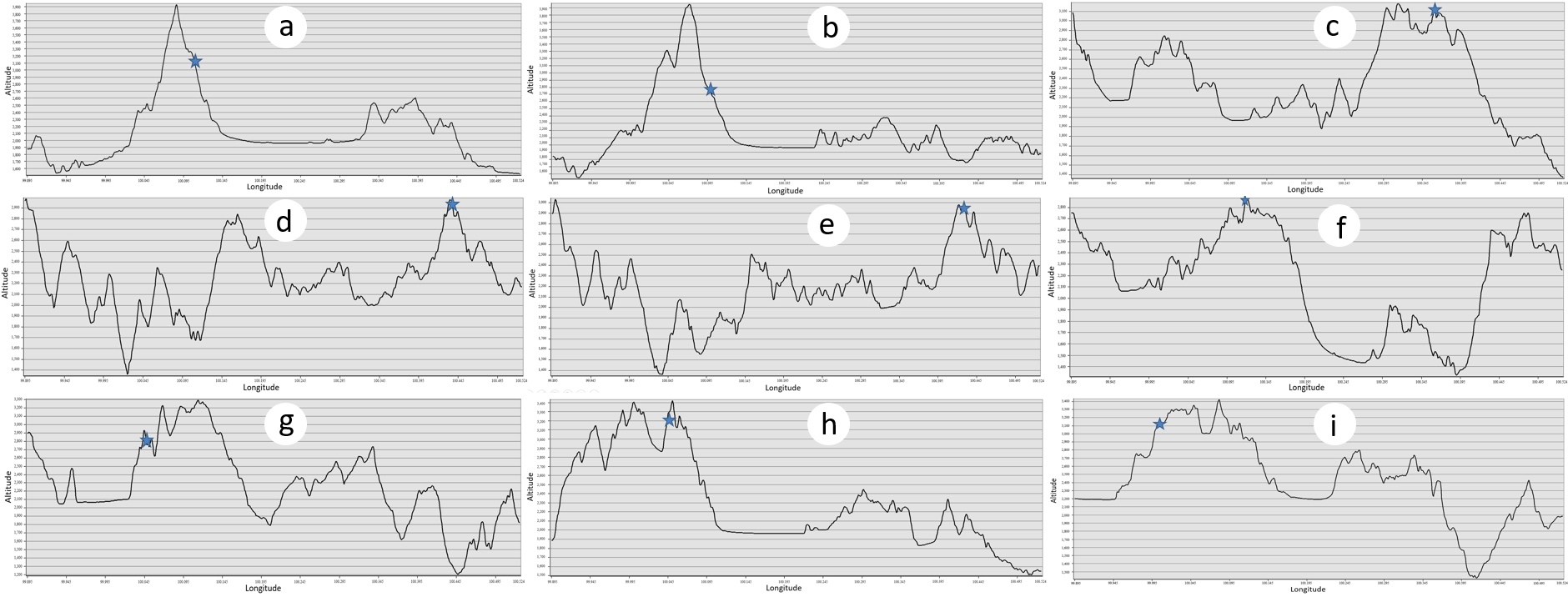

Supplement: Supplementary file 11 — Fig.S2 [file 41438_2020_407_MOESM11_ESM.jpg]

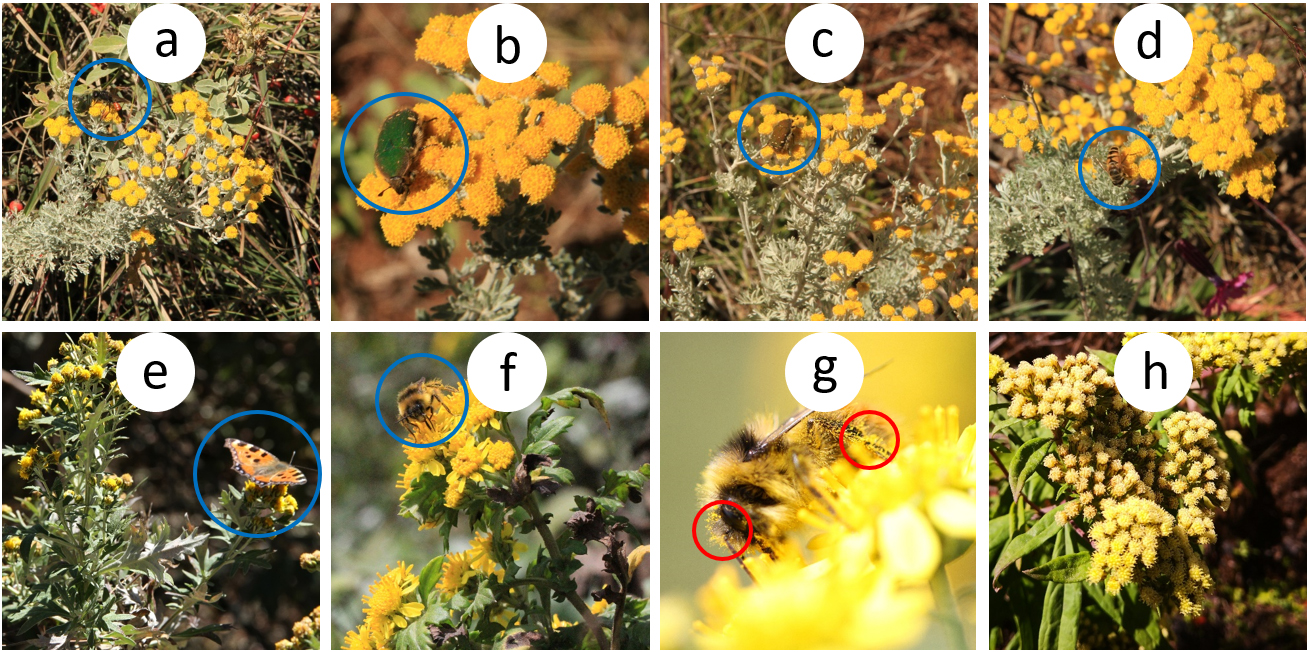

Supplement: Supplementary file 12 — Fig.S3 [file 41438_2020_407_MOESM12_ESM.jpg]

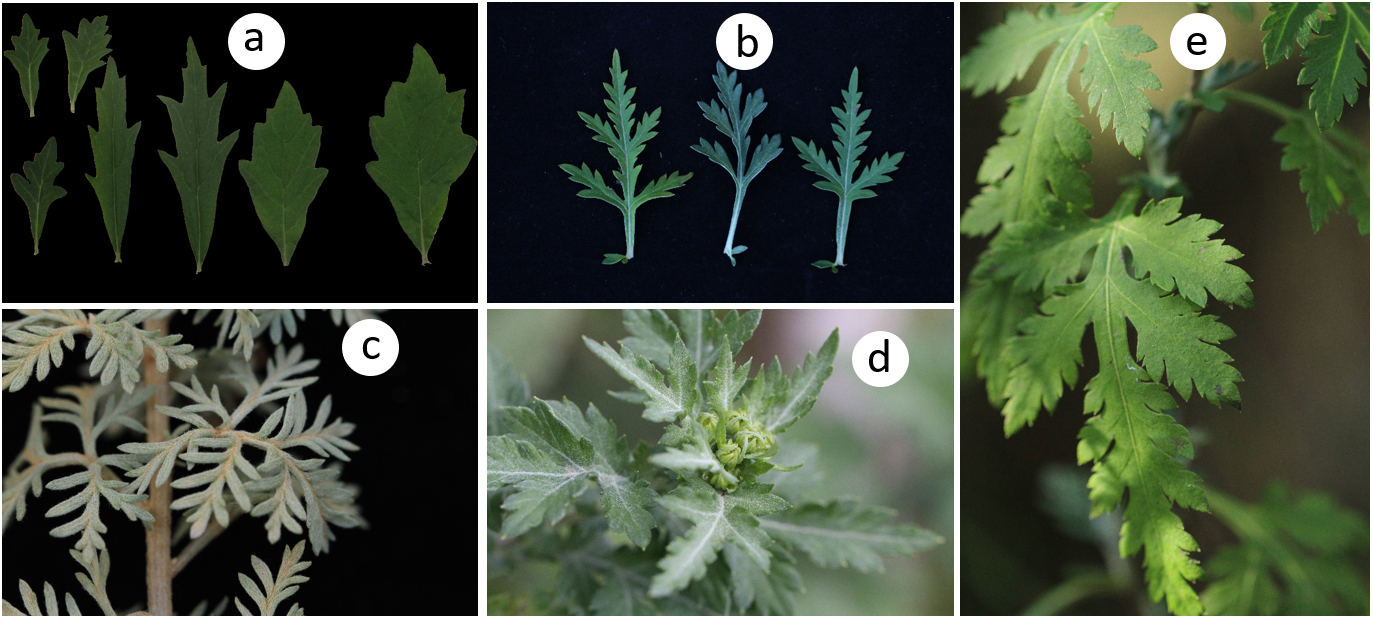

Supplement: Supplementary file 13 — Fig.S4 [file 41438_2020_407_MOESM13_ESM.jpg]

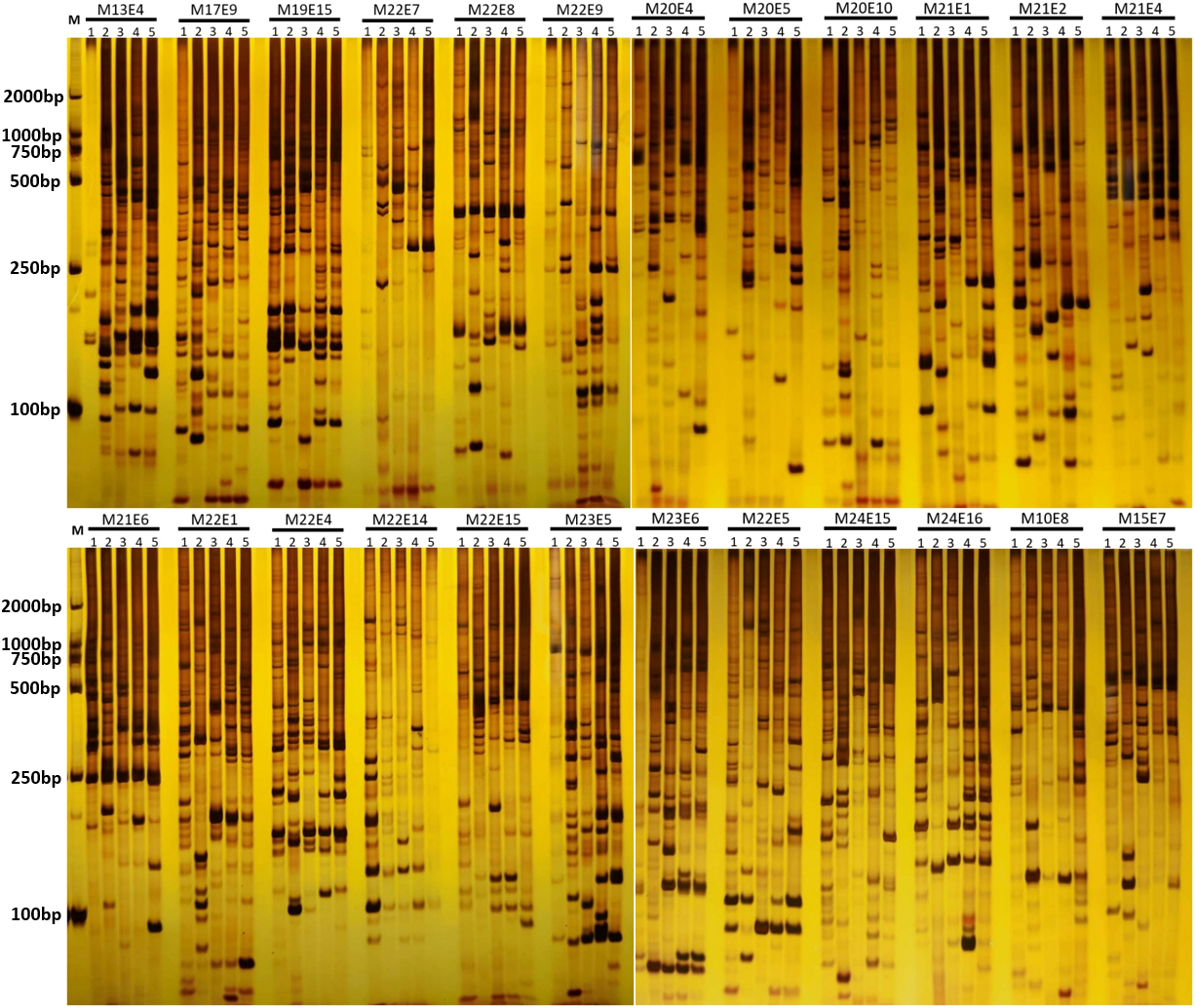

Supplement: Supplementary file 14 — Fig. S5 [file 41438_2020_407_MOESM14_ESM.jpg]
